# A data platform to improve rabies prevention, Sri Lanka

**DOI:** 10.2471/BLT.16.188060

**Published:** 2017-05-19

**Authors:** A Pubudu De Silva, PA Lionel Harischandra, Abi Beane, Shriyananda Rathnayaka, Ruwini Pimburage, Wageesha Wijesiriwardana, Dilanthi Gamage, Desika Jayasinghe, Chathurani Sigera, Amila Gunasekara, Mizaya Cadre, Sarath Amunugama, Priyantha L Athapattu, K Saroj A Jayasinghe, Arjen M Dondorp, Rashan Haniffa

**Affiliations:** a Network for Improving Critical Care Systems and Training, 2nd Floor, YMBA Building, Colombo 08, Sri Lanka.; b Public Health Veterinary Services, Colombo, Sri Lanka.; c Mahidol Oxford Tropical Medicine Research Unit, Bangkok, Thailand.; d Information Communication Technology Agency of Sri Lanka, Colombo, Sri Lanka.; e National Hospital of Sri Lanka, Colombo, Sri Lanka.; f Office of Deputy Director General (Public Health Services) I, Ministry of Health, Colombo, Sri Lanka.; g Office of Director Medical Services, Ministry of Health, Colombo, Sri Lanka.; h Faculty of Medicine, University of Colombo, Colombo, Sri Lanka.

## Abstract

**Problem:**

In Sri Lanka, rabies prevention initiatives are hindered by fragmented and delayed information-sharing that limits clinicians’ ability to follow patients and impedes public health surveillance.

**Approach:**

In a project led by the health ministry, we adapted existing technologies to create an electronic platform for rabies surveillance. Information is entered by trained clinical staff, and both aggregate and individual patient data are visualized in real time. An automated short message system (SMS) alerts patients for vaccination follow-up appointments and informs public health inspectors about incidents of animal bites.

**Local setting:**

The platform was rolled out in June 2016 in four districts of Sri Lanka, linking six rabies clinics, three laboratories and the public health inspectorate.

**Relevant changes:**

Over a 9-month period, 12 121 animal bites were reported to clinics and entered in the registry. Via secure portals, clinicians and public health teams accessed live information on treatment and outcomes of patients started on post-exposure prophylaxis (9507) or receiving deferred treatment (2614). Laboratories rapidly communicated the results of rabies virus tests on dead mammals (328/907 positive). In two pilot districts SMS reminders were sent to 1376 (71.2%) of 1933 patients whose contact details were available. Daily SMS reports alerted 17 public health inspectors to bite incidents in their area for investigation.

**Lessons learnt:**

Existing technologies in low-resource countries can be harnessed to improve public health surveillance. Investment is needed in platform development and training and support for front-line staff. Greater public engagement is needed to improve completeness of surveillance and treatment.

## Résumé

**Problème:**

Au Sri Lanka, les initiatives de prévention de la rage sont entravées par une diffusion des informations fragmentaire et lente, ce qui limite la capacité des cliniciens à suivre les patients et empêche la mise en œuvre d'une surveillance sanitaire.

**Approche:**

Dans le cadre d'un projet supervisé par le ministère de la Santé, nous avons adapté les technologies existantes afin de créer une plateforme électronique pour la surveillance de la rage. Les informations y sont saisies par du personnel clinique formé, et les données de chaque patient ainsi que les données globales y sont accessibles en temps réel. Un service automatisé de minimessages (SMS) avertit les patients à l'approche de leurs rendez-vous de suivi et de vaccination de rappel et informe les inspecteurs de santé publique des nouveaux cas de morsures d'animaux.

**Environnement local:**

La plateforme a été mise en œuvre en juin 2016 dans quatre districts du Sri Lanka. Elle relie six centres antirabiques, trois laboratoires et l'inspection de la santé publique.

**Changements significatifs:**

En 9 mois, 12 121 cas de morsures d'animaux ont été pris en charge par les centres antirabiques et saisis dans le registre. Les cliniciens et les équipes de santé publique ont pu accéder, via des portails sécurisés, à des informations actualisées en direct sur les traitements et l'évolution des patients, suivis dans le cadre d'un traitement prophylactique post-exposition (9 507) ou d'un traitement retardé (2 614). Les laboratoires ont pu communiquer rapidement les résultats des tests de dépistage de la rage réalisés sur des mammifères morts (328 cas positifs sur 907). Dans deux districts pilotes correspondant à un total de 1 933 patients pris en charge, des rappels par SMS ont été envoyés aux 1 376 patients (71,2%) dont les coordonnées de contact étaient connues. Des rapports SMS quotidiens ont permis d'avertir 17 inspecteurs de santé publique de nouveaux cas de morsures survenus dans leur secteur, pour enquête.

**Leçons tirées:**

Il est possible d'exploiter les technologies existantes, dans les régions à faibles ressources, pour améliorer la surveillance de la santé publique. Il est encore nécessaire d'investir dans le développement de cette plateforme et dans la formation et l'assistance du personnel de première ligne. Une plus grande sensibilisation du public est également nécessaire pour améliorer l'exhaustivité de la surveillance et des traitements.

## Resumen

**Situación:**

En Sri Lanka, las iniciativas de prevención de la rabia se ven obstaculizadas por un intercambio de información fragmentado y retrasado que limita la capacidad de los médicos de hacer seguimientos a sus pacientes e impide el control de la atención sanitaria.

**Enfoque:**

En un proyecto dirigido por el ministerio de sanidad, se adaptaron las tecnologías existentes para crear una plataforma electrónica para el control de la rabia. Personal clínico formado introduce la información, y los datos sobre pacientes se pueden consultar en tiempo real tanto de forma individual como global. Un sistema automatizado de mensajes cortos (SMS) alerta a los pacientes de citas para vacunas de seguimiento e informa a los inspectores de salud pública sobre incidentes de mordeduras de animales.

**Marco regional:**

La plataforma se implementó en junio de 2016 en cuatro distritos de Sri Lanka y vinculó seis clínicas de atención contra la rabia, tres laboratorios y el cuerpo de inspectores de salud pública.

**Cambios importantes:**

En un periodo de 9 meses, se informó a las clínicas de 12 121 mordeduras de animales, las cuales fueron inscritas en el registro. A través de portales seguros, los médicos y los equipos de salud pública accedieron a información en directo sobre el tratamiento y los resultados de los pacientes que comenzaron con profilaxis posterior a la exposición (9 507) o con tratamiento aplazado (2 614). Los laboratorios comunicaron con rapidez los resultados de las pruebas del virus de la rabia en animales muertos (328/907 positivos). En dos distritos piloto, se enviaron recordatorios SMS a 1 376 (71,2%) de los 1 933 pacientes de los que se disponían detalles de contacto. Informes con SMS diarios alertaron a 17 inspectores de salud pública sobre incidentes de mordeduras en su zona para que los investigaran.

**Lecciones aprendidas:**

Las tecnologías existentes se pueden aprovechar en países con pocos recursos para mejorar el control de la salud pública. Se necesita invertir en el desarrollo de plataformas y en la formación y el apoyo del personal de primera línea. Es necesario un mayor compromiso público para mejorar la finalización del control y el tratamiento.

## ملخص

**المشكلة:**

تواجه مبادرات الوقاية من داء الكَلْب صعوبات في سرى لانكا بسبب التشتت والتأخير في مشاركة المعلومات، الأمر الذي يحد من قدرة الأطباء على متابعة المرضى كما يعوق رصد الصحة العامة.

ا **لأسلوب **قمنا بتهيئة التقنيات القائمة لإنشاء منصة إلكترونية لمراقبة داء الكَلْب في إطار مشروع قادته وزارة الصحة. ويتولى أفراد الطاقم السريري المدربون إدخال المعلومات، فيما يتم تقديم عرض مرئي لكلٍ من بيانات المرضى المجمعة والبيانات الفردية في الوقت الفعلي. كما يقوم نظام الرسائل الآلية القصيرة (SMS) بتنبيه المرضى بمواعيد متابعة التطعيم وإبلاغ مفتشي الصحة العامة عن حوادث عضات الحيوانات.

**المواقع المحلية:**

تم طرح المنصة للعمل في شهر حزيران/يونيو عام 2016 في أربعة أحياء في سرى لانكا، مما ساعد في ربط ست عيادات لمعالجة داء الكَلْب وثلاثة مختبرات فضلاً عن هيئة التفتيش على الصحة العامة.

**التغييرات ذات الصلة:**

على مدى فترة 9 أشهر، وردت بلاغات عن 12,121 حالة من عضات الحيوانات إلى العيادات وتم إدخالها في السجل. ومن خلال البوابات الإلكترونية الآمنة، فقد تمكن الأطباء وفرق الصحة العامة من الوصول إلى معلومات مباشرة عن العلاج ونتائج المرضى الذين بدأوا الوقاية المرض بعد التعرض للإصابة (9507) أو الذين بدأوا العلاج المؤجل (2614). وسرعان ما أبلغت المختبرات عن نتائج اختبارات فيروس داء الكَلْب على الثدييات الميتة (‏328/907 إيجابية). وفي منطقتين تجريبيتين، تم إرسال رسائل تذكيرية إلى 1376 (71.2‏٪) من أصل 1933 مريضًا توفرت تفاصيل الاتصال بهم. كما نبهت التقارير اليومية عبر الرسائل القصيرة 17 مفتشًا للصحة العامة عن حوادث لدغ في مناطق اختصاصهم لإجراء التحقيق فيها.

**الدروس المستفادة:**

يمكن تسخير التقنيات القائمة في البلدان منخفضة الموارد لتحسين مراقبة الصحة العامة. كما يلزم الاستثمار في المنصة لتطوير وتدريب ودعم العاملين في الخطوط الأمامية. وهناك حاجة إلى مزيد من المشاركة العامة لتحسين إتمام عمليات الرصد والعلاج.

## 摘要

**问题:**

在斯里兰卡，狂犬病预防措施因分散和延迟的信息共享而受到阻碍，这也限制了临床医生追踪患者的能力，并且妨碍了公共卫生监督。

**方法:**

在一个卫生部领导的项目中，我们对现有技术进行了调整，建立了一个狂犬病监测电子平台。 由经过培训的临床人员输入信息，并且会实时显示总体和个体患者数据。 自动化短信系统 (SMS) 可通知患者进行后续的疫苗接种，并向公共卫生检查员通报动物咬伤事件。

**当地状况:**

该平台于 2016 年 6 月在斯里兰卡的四个地区推出，连接六个狂犬病诊所、三个实验室和相应公共卫生检查员。

**相关变化:**

在 9 个月的期间，共向诊所报告并登记在册 12 121 例动物咬伤事件。 通过安全门户网站，临床医生和公共卫生团队访问了从接触后预防开始治疗 (9507) 或接受延迟治疗 (2614) 的患者的治疗和结果实时信息。 实验室迅速传达了死亡哺乳动物狂犬病病毒检测的结果（328/907 阳性）。 在两个试点地区，向 1933 名提供了详细联系信息的患者中的 1376 (71.2%) 发送了短信提醒。 每日短信报告向 17 名公共卫生检查员通报其区域内发生的咬伤事件以进行调查。

**经验教训:**

可利用低资源国家的现有技术改善公共卫生监督。 需要在平台开发以及一线员工的培训和支持方面进行投资。 需要更多的公众参与以完善监督和治疗的完整性。****

## Резюме

**Проблема:**

В Шри-Ланке инициативам по профилактике бешенства препятствует фрагментированный и медленный обмен информацией, который ограничивает возможности врачей по наблюдению за пациентами и препятствует надзору за общественным здравоохранением.

**Подход:**

В рамках проекта, возглавляемого Министерством здравоохранения, мы адаптировали существующие технологии для создания электронной платформы наблюдения за бешенством. Информация вводится обученным клиническим персоналом, при этом как совокупные, так и индивидуальные данные о пациенте визуализируются в реальном времени. Автоматизированная система коротких сообщений (SMS) предупреждает пациентов о назначении последующих прививок и информирует инспекторов общественного здравоохранения о случаях нападения животных на людей.

**Местные условия:**

Платформа была внедрена в июне 2016 года в четырех районах Шри-Ланки, объединив шесть клиник бешенства, три лаборатории и инспекцию общественного здравоохранения.

**Осуществленные перемены:**

За 9-месячный период в клиниках был зарегистрирован и внесен в реестр 12 121 случай укусов со стороны животных. Через защищенные порталы медицинские работники и группы общественного здравоохранения получили доступ к актуальной информации о лечении и исходах пациентов, начавших постконтактную профилактику (9507) или пациентов с отсроченным лечением (2614). Лаборатории быстро сообщали результаты тестов на вирус бешенства у мертвых млекопитающих (328 положительных из 907). В двух экспериментальных районах SMS-напоминания были отправлены 1376 (71,2%) из 1933 пациентов, чьи контактные данные были доступны. Ежедневные SMS-отчеты предупреждали 17 инспекторов здравоохранения о случаях нападения животных на людей в их районе для проведения контроля.

**Выводы:**

Существующие технологии в странах с низким уровнем ресурсов могут быть использованы для улучшения эпиднадзора за общественным здравоохранением. Необходимы инвестиции в разработку платформы, обучение и поддержку обслуживающего персонала. Для улучшения полноты эпиднадзора и лечения требуется более активное участие общественности.

## Introduction

As in many other countries in Africa and Asia,^1^ rabies remains a public health problem in Sri Lanka. In 2015, 607 of 1166 animal heads tested positive for rabies and 24 human deaths were reported.^2^

In 1975 the Sri Lankan Ministry of Health initiated a series of nationwide rabies prevention measures, coordinated by the public health veterinary services. These covered the introduction of dog vaccination (including stray dogs after 1997), post-exposure prophylaxis for patients presenting after an animal bite (after 1992) and sterilization of stray dogs (after 2008).^3^ In 2015 1 294 529 domestic dogs and 152 404 stray dogs were vaccinated and 274 405 patients started on post-exposure prophylaxis.^2^ Patients who are bitten by a mammal can attend rabies clinics in government hospitals where post-exposure prophylaxis is provided free of charge. Public health information ensures high public awareness of rabies risk and the location of clinics. If the bite is low severity or the animal is known, treatment is deferred for 14 days while the animal is observed for clinical signs of rabies.^4^ Otherwise, post-exposure prophylaxis is started, which requires the patient to attend the clinic for vaccination with inactivated cell-culture-based rabies vaccine on days 0, 3, 7 and 30 following presentation.^5^ Samples of dead animals from all parts of Sri Lanka are sent to specialist laboratories that use direct fluorescent antibody testing for the presence of rabies virus.

However, clinical treatment, prevention initiatives and surveillance of rabies epidemiology in Sri Lanka are hindered by weaknesses in record-keeping and data collection. Clinic health records and reports to the public health inspectorate are paper-based and regional variations in completeness of record-keeping exist. Monthly reports are collated and disseminated in an electronic database. Clinicians therefore have limited access to information on a patient’s completion of post-exposure prophylaxis regimes and nationwide surveillance to ascertain the completion rate of anti-rabies treatment in Sri Lanka is poor.^6^ Similarly, there is no system for contacting patients to attend for vaccinations. This contributes to missed, delayed or duplicated vaccinations.^7^^,^^8^ Another problem is that feedback of laboratory results to the public health veterinary services is via manual monthly reports. This delays the ability of front-line teams to identify and respond to rabies outbreaks, locate infected animals and contact patients associated with animals that have tested positive. Finally, public health inspectors and the public health veterinary services have limited ability to evaluate the effectiveness of the current canine vaccination programme on a national basis and to identify regional variations in activity (e.g. urban compared with rural locations) and transmission characteristics.^1^

This paper describes the creation of an electronic platform to improve the collection and sharing of data for better management and surveillance of rabies in Sri Lanka. The platform was developed by adapting existing acute and critical care platforms^9^ and was driven by clinicians looking for solutions to the problems outlined above.

## Approach

The project was a joint initiative of the public health veterinary services of the health ministry and the Network for Improving Critical Care Systems and Training Sri Lanka. It aimed to provide real-time data on: (i) the incidence and characteristics of mammal bites to humans; (ii) patient demographics; (iii) treatment decisions and patient outcomes; and (iv) the prevalence of rabies in dead mammals.

The design of the data platform was clinician-led, focusing on the information needed to improve front-line care. We conducted scoping interviews with public health veterinary services and clinical staff to determine users’ data requirements. In hospitals the required variables included patient characteristics, animal characteristics, severity of the bite and patient treatment details (Box 1). In laboratories the variables included type of animal, habitat, mode of death, vaccination status and investigation results. We developed the data collection tool using REDCap software (REDCap Consortium, Nashville, United States of America). The tool was accessible to users via the internet from desktop computers and mobile devices. Data on each bite incident are entered by trained nursing officers when patients first present to a hospital rabies clinic. Data on animal test results are entered by clinical staff in the relevant laboratories.

Box 1 **Information available on dashboards of the electronic platform for improving surveillance and treatment data on rabies in Sri Lanka, 2016**

**Overview category**

- Bite incident details: geographical location, date of incident, bite severity (major or minor), mammal classification, vaccination summary, status of mammal (dead or alive).
- Bite incident reporting rates of hospitals and public health inspectors.
- Patient’s demographic and clinical data: name, age, sex, occupation, date of presentation to the hospital, bite severity, vaccination dates and completeness.
- Treatment: physician’s decision (treatment initiated or deferred), anti-rabies virus vaccine treatment regime (days 0, 3, 7 & 30), treatment completeness (including delays in vaccine doses).
- Laboratory investigations: investigation type (FAT, ICT, MIT, PCR), status of mammal (dead or alive), test result (rabies virus positive or negative), geographical location of rabies-positive animal.

**Treatment follow-up category**

- Patient’s demographic and clinical data: contact details, bite incident details (date, mammal, severity), treatment (treatment initiated or deferred).
- Patient’s appointment attendance (attended or not).
- Patient’s anti-rabies virus vaccine treatment status (vaccinated, not vaccinated, yet to vaccinate or observation of animal only).

**Laboratory testing category**

- Patient’s demographic data, bite incident details, physician’s treatment decisions.
- Biopsy test results (positive or negative), by area.
- Classification of animal (dog, cat, domestic-other, non-domestic-other).

FAT: fluorescent antibody test; ICT: immunochromatographic test strip; MIT: mouse inoculation test; PCR: polymerase chain reaction.

Initial training was done by the central registry team at visits to rabies clinics and laboratories. During the implementation phase, ongoing clinician support was provided by the registry staff by telephone, with daily validation phone calls to the clinics to determine the numbers and motivate staff towards data entry. Laboratories were also contacted daily by the central registry validation team during implementation to help improve information completeness and staff skills.

We used a business analytics software (Tableau version 10.0, Tableau Software, Seattle, USA) to develop dashboards that would enable users to visualize the data in real-time, either as an aggregate or filtered by incident location or characteristics. The data are accessible to front-line staff (nurses and doctors), administrators, public health inspectors and laboratory teams via an online portal. Aggregated data are accessible to the general public, so that trends in bite incidence, patient demographics, mammal characteristics and laboratory test results can be identified. Individual patient data and laboratory results require a two-step authentication and are accessible only to clinicians treating the patient and to public health inspectors. The data are then displayed through a dashboard via the registry platform in the secure servers of the Information and Communication Technology Agency of the Government of Sri Lanka.^10^

**Fig. 1:**
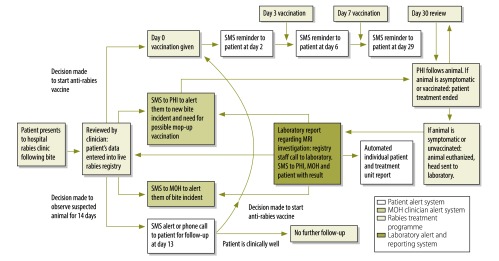
Schematic overview of the real-time SMS alert system for animal-bitten patients and health-care teams in Sri Lanka

MOH: Ministry of Health; MRI: magnetic resonance imaging; PHI: public health inspectorate; SMS: short message system.

Mobile phone ownership is very high in Sri Lanka and follow-up of patients is aided by linking the post-exposure prophylaxis dashboard to a bespoke automated short message system (SMS). The clinician enters the patient’s mobile phone number and national identification number (the only unique identifier) to enable treatment episodes and outcomes to be linked. SMS alerts are sent to the patient the day before the due date of anti-rabies vaccine. Patients with deferred treatment are contacted on day 14 of the observation period of the suspected animal. Teams in rabies clinics are alerted by SMS to the numbers of patients due for anti-rabies vaccine each day, while public health inspectors are alerted to the incidence and characteristics of bites reported within their region, and any subsequent animal deaths. A schematic overview of the platform is shown in Fig. 1.

## Relevant changes

The live platform was rolled out on 1 June 2016 across four districts in Sri Lanka (two urban and two rural: Colombo, Gampaha, Mathale and Monaragala), with a combined population of 3 224 923. It linked six hospital clinics, three laboratories (Medical Research Institute, Karapitiya and Peradeniya) and the public health inspectorate. The SMS alert system was initiated on 1 September 2016 in two of the districts that volunteered to participate.

Between 1 June 2016 and 28 February 2017, 12 121 reported incidents of animal bites from the four districts were entered into the registry, providing real-time data on the incidence, character and geographical location of mammal bites. Clinicians and public health teams were able to access data on the clinical management and outcomes of patients on post-exposure prophylaxis (9507; 78.4%) or with treatment deferred (2614; 21.6%). Five of the six clinics reported on the completeness of post-exposure prophylaxis, showing that 293 (7.9%) of 3709 patients completed all four anti-rabies vaccinations. Data on other patient outcomes (e.g. delays in starting treatment, number of deaths) will be available in the future. Information on the characteristics of suspected sources (60.8% dogs, 24.8% cats, 7.1% rats and other 7.4% non-domesticated mammals) enabled the public health veterinary services to evaluate current animal vaccination strategies and identify regional epidemiological variances.

Of 907 dead mammals tested, 328 (36.2%) were positive for rabies virus. The laboratories entered these data into the registry so that animal test results could be communicated rapidly to the clinic teams and the public health inspectorate responsible for individual cases.

Over the 6-month period, the SMS alert system in two districts alerted 1376 (71.2%) patients on post-exposure prophylaxis to attend their vaccination appointments; contact details were unavailable for 557 patients. Daily SMS reports alerted 17 public health inspectors to 3125 bite incidents in their region for further investigation.

## Lessons learnt

This proof-of concept study illustrates how technologies that already exist in many low- and middle-income countries can be harnessed with existing staff resources to create an electronic platform for improving public health surveillance (Box 2). Maximizing the availability of information on the incidence, geographical location and outcomes of mammal bites should facilitate timely responses to incidents, and inform decisions about resource allocation and the refinement of treatment and prevention programmes. The SMS alerts health-care responders to clusters of incidents in their region and aims to improve compliance by patients on treatment; similar systems have proved effective in other health-care settings.^11^ Work is ongoing with front-line staff to improve completeness of contact information and to explore low treatment compliance in this setting.

Box 2 **Summary of main lessons learnt**

Existing technologies in low- and middle-income countries can be harnessed using minimal resources to provide a platform for improved public health surveillance.

To expand the platform within Sri Lanka and to test its transferability to other settings, investment is needed in platform development and training and support for front-line staff.

Greater public engagement is needed to improve completeness of surveillance and treatment.

To expand the platform within Sri Lanka, investment is needed in platform development and in training and support for clinical staff, both to address data completeness and to refine the platform before scaling-up the initiative. Such investment would also allow an evaluation of the impact of the platform on reducing avoidable delays in starting post-exposure prophylaxis. It would also inform targeted animal vaccinations, using the data on where and when bites occurred. The existing media campaign may need to be boosted to raise the public’s awareness of the importance of reporting animal bites and complying with post-exposure care.

The next stage is to extend the platform to all rabies clinics nationwide to assist clinicians and administrators throughout the country to organize resources and improve the completeness rates of post-exposure prophylaxis. This information could be accessed remotely by specialists to help local teams refine prevention strategies based on regional epidemiological information and to enable clinicians to evaluate existing and future treatment protocols for rabies and other communicable diseases.
